# Electrophysiological characterization of entopeduncular nucleus neurons in anesthetized and freely moving rats

**DOI:** 10.3389/fnsys.2014.00007

**Published:** 2014-02-10

**Authors:** Liora Benhamou, Dana Cohen

**Affiliations:** ^1^The Leslie and Susan Gonda Multidisciplinary Brain Research Center, Bar-Ilan UniversityRamat-Gan, Israel

**Keywords:** basal ganglia, electrophysiology, anesthesia, extracellular recording, firing patterns, neuronal population

## Abstract

The EntoPeduncular nucleus (EP), which is homologous to the internal segment of the Globus Pallidus (GPi) in primates, is one of the two basal ganglia (BG) output nuclei. Despite their importance in cortico-BG information processing, EP neurons have rarely been investigated in rats and there is no available electrophysiological characterization of EP neurons *in vivo*. We recorded and analyzed the activity of EP neurons in freely moving as well as anesthetized rats, and compared their activity patterns. Examination of neuronal firing statistics during wakefulness suggested that similar to neurons recorded in the primate GPi, EP neurons are a single population characterized by Poisson-like firing. Under isoflurane anesthesia the firing rate of EP neurons decreased substantially and their coefficient of variation and relative duration of quiescence periods increased. Investigation of the relationship between firing rate and depth of anesthesia revealed two distinct neuronal groups: one that decreased its firing rate with the increase in anesthesia level, and a second group where the firing rate was independent of anesthesia level. *Post-hoc* examination of the firing properties of the two groups showed that they were statistically distinct. These results may thus help reconcile *in vitro* studies in rats and primates which have reported two distinct neuronal populations, and *in vivo* studies in behaving primates indicating one homogeneous population. Our data support the existence of two distinct neuronal populations in the rat EP that can be distinguished by their characteristic firing response to anesthesia.

## Introduction

The Basal Ganglia (BG), a group of subcortical nuclei, process and integrate motor, cognitive, and limbic information arriving from most cortical areas and the thalamus via a network of segregated pathways (Alexander et al., 1986; Alexander and Crutcher, 1990; Deniau et al., 1996; Kitano et al., 1998). The globus pallidus internal segment (GPi), which is one of the two output stations of the BG, projects to the cortex through different thalamic nuclei, and influences information processing in these targets (Kravitz et al., 2010; Kojima et al., 2013). The GPi is thought to participate in motor control especially in movement initiation, sequential organization of movement and action selection (Horak and Anderson, 1984; Mink, 1996; Redgrave et al., 1999). GPi lesions cause motor disturbances such as reduced speed and acceleration, resulting in hypometria (Turner and Anderson, 2005; Turner and Desmurget, 2010). By contrast, ablation of the GPi has been shown to improve the conditions of patients suffering from Parkinson’s disease, which suggests that abnormal information processing in the GPi may have a greater influence on motor behavior than its silencing (Obeso et al., 2009). Additionally, it has been shown that GPi neurons process sensory information related to reward consumption (Lidsky, 1975), and that they are also tuned to reward predicting targets (Hong and Hikosaka, 2008), reward probability (Joshua et al., 2009) and that their activity facilitates response variability required for associative learning (Sheth et al., 2011). Moreover, it has been suggested that the GPi neurons may initiate reward related signals through their effect on the lateral habenula (LHb) (Hong and Hikosaka, 2008). Overall, the complexity of BG information processing encourages further examination of information flow through the different BG nuclei.

Primate GPi neurons are anatomically and biochemically comparable to the neurons of the GPe, a central station of the BG. However, extracellular electrophysiological characterizations of the firing properties of GPe neurons suggest there are two distinct neuronal groups of neurons dubbed high frequency pausers and low frequency bursters, whereas a similar analysis of GPi neurons reveals only one neuronal population (DeLong, 1971). GPi neurons display continuous high rate discharges of about 60 spikes/s with rapid firing rate fluctuations ranging from 10 to 107 spikes/s but without displaying pauses or prolonged silences as is the case for GPe neurons (DeLong, 1971). Other studies have reported that GPi neurons display uncorrelated non-synchronous activity (Bar-Gad et al., 2003a) and that they exhibit slow oscillations in the multisecond range (Wichmann et al., 2002). Currently there is no established characterization of neuronal activity in EntoPeduncular (EP) neurons in freely moving rats.

In general, the rodent and primate BG share similar cell types and connectivity. Thus comparative studies may help clarify the nature, role and features of the computation taking place in these structures. We previously compared the rat Globus Pallidus (GP) activity to the GPe, its primate homologue (Benhamou et al., 2012) and showed that like the primate GPe, the rat GP comprised two neuronal populations. Although the neuronal firing rates in the GP were substantially lower than those observed in the GPe, both species displayed similar firing pattern characteristics. Since the EP is homologous to the primate GPi, it was hypothesized that an extracellular electrophysiological characterization of the firing properties of EP would reveal a single population. Contrary to this assumption, however, anatomical tracing studies in primates, cats, and rats have reported that based on their projection target sites, it is likely that the GPi/EP neurons form two neuronal populations (Nauta, 1974; Filion and Harnois, 1978; van der Kooy and Carter, 1981; Tokuno et al., 1988; Parent et al., 2001). These anatomical studies were corroborated by a study in slice preparation of the rat EP that described two distinct neuronal populations based on their intrinsic electrical properties (Nakanishi et al., 1990).

Here, we characterize and compare neuronal activity recorded chronically in the EP of freely moving rats during wakefulness and under different levels of isoflurane anesthesia. Considering the similarity in the electrophysiological properties of GPe neurons between rats and primates (Benhamou et al., 2012) we hypothesized that rat EP neurons would display similar properties to those observed in primate GPi. During wakefulness, the recorded neurons displayed tonic activity with near Poissonian firing. The distributions of neuronal statistics such as firing rate (FR), coefficient of variation (CV), fano factor (FF) and relative duration of quiescence periods (Silence Index) calculated in freely moving animals were spread along a continuum, thus suggesting that this sample was composed of one neuronal population. By contrast, the characterization of neuronal activity measured under different levels of anesthesia revealed two distinct groups: one that decreased firing rate correlatively with anesthesia level and a second whose firing rate was independent of anesthesia. *Post-hoc* examination of the two population statistics revealed that despite being spread over a continuum, the characteristics of the two populations were significantly different. Our data thus suggest the existence of two neuronal populations in the rat EP that can only be distinguished under anesthesia.

## Materials and Methods

All procedures were carried out in accordance with the National Institutes of Health Guide for the Care and Use of Laboratory Animals and the Bar-Ilan University Guidelines for the Use and Care of Laboratory Animals in Research.

### Surgical procedure

The surgical procedure was previously described (Nicolelis et al., 1997; Jacobson et al., 2009). In brief, four adult male Long-Evans rats (Harlan) weighing 450 g on average (range: 430–480 g) were sedated with 5% isoflurane and were then injected i.m. with ketamine HCl and xylazine HCl (100 and 10 mg/kg, respectively). Supplementary injections of xylazine and ketamine were administrated if required. Rats were maintained by a stereotaxic frame (Kopf Instruments, USA). After sterilization, the rat’s head was incised and the skull surface revealed. Connective tissue was then removed and the skull surface cleaned. Two bilateral craniotomies slightly larger than the electrode were made above the EP (anteroposterior (AP): 2.4 mm, mediolateral (ML): 3.0 mm, dorsoventral (DV): −7.0 mm). Eight Formvar coated Nichrome wires (coated diameter: 0.0015″, A-M Systems, Inc.) placed in a 27 Gauge cannulae were slowly introduced into the EP through each craniotomy independently. Electrodes (impedance 0.1–0.2 MΩ at 1 kHz) were cemented in place with dental acrylic, leaving the upper part of the connectors exposed.

At the end of experiment, the rats were anesthetized with 5% isoflurane, then ketamine, xylazine and morphine (3 mg/Kg) were injected i.p and electrolytic lesions made before perfusion with 4% formaldehyde. The brain was fixated with 20% sucrose and sectioned with cryostat in 60 μm slices. Electrode positions were confirmed histologically using a microscope (NikonEclipse E400, 1x/400).

### Data collection

Following about 10 days of recovery from surgery, the activity of EP neurons was recorded in four adult male Long-Evans rats. Within each session the rats were anesthetized with gradually decreasing doses of isoflurane (4% to 1% by steps of 1%) followed by a period of wakefulness during which the rats moved around in the recording chamber rather than remained immobile. Based on our observations of the elapsed time from anesthesia removal until animals started to walk freely in the recording chamber we estimated a recovery time of 5 min for each alteration in isoflurane level thus allowing neuronal activity to physiologically adjust to the different levels. Hence, data collection started after a 5 min intermission following each transition in anesthesia depth. Neural activity was amplified, band-pass filtered at 150–8000 Hz and sampled at 40 kHz using a multichannel acquisition processor system (MAP system; Plexon Inc, Dallas, TX, USA). Offline sorting was performed on all continuously recorded units (OfflineSorter V2.8.8; Plexon, Dallas, TX) and the data were analyzed using custom-written MATLAB software (R2010b, MathWorks Inc., Natick, MA).

## Data analysis

### Statistical analysis

All the data reported are presented as the mean ± SEM. Unless stated otherwise, statistically significant differences between parameters were assessed by the non-parametric test Kruskal-Wallis with the Tukey-Kramer multiple comparison correction.

In order to identify significant nonlinear correlation between firing rate and isoflurane, we used the a-parametric Kendall-Tau correlation test (Matlab function: corr with type Kendall; *p* < 0.05). The Kendall-tau test provides a coefficient that represents the degree of concordance between two columns of ranked data. It is similar to the non-parametric Spearman’s *ρ*-test but it enables easier interpretation of the correlation value. Specifically, it is the difference between the probability that the observed data are in the same order and the probability that the observed data are not in the same order.

### Waveform parameters

The waveform parameters consisted of the valley to peak ratio, valley to peak duration, valley width, zero-cross, and peak and valley amplitudes. The valley is the minimal amplitude time point and the peak is the first maximum observed after the valley time point. Valley width quantifies the duration of the extracellular waveform at its half amplitude, the valley to peak ratio is the valley amplitude divided by the absolute value of the peak amplitude, and zero-cross is the time elapsed between the two time points around the valley in which the amplitude equals zero (see inset in Figure 3A).

### Firing parameters

The firing parameters included the CV, FF, FR, refractory period and mode Inter-Spike Interval (ISI). The term CV defines the standard deviation of the ISI distribution divided by its mean. The FF is the variance of the spike count distribution calculated in non-overlapping time windows, divided by its mean (window duration equals the median ISI of each neuron). The FR is the total number of spikes divided by the total recording time (spike count rate). The refractory period defines the time in ms elapsed from 0 on the neurons’ auto-correlogram to attain its half height at stable state. Mode ISI describes the mode value of the ISI distribution using 5 ms precision bins.

### Auto- and cross-correlations

Autocorrelation and crosscorrelation functions were calculated for latencies of 1000 ms and 500 ms respectively (bin size equals 1 ms).

For the cross-correlation functions, upper and lower confidence levels were calculated as follows: the mean and standard deviation of the cross-correlograms at time ±4–5 s were calculated. The probability that the signal crossed a specified limit in 1% of the bins over 1 s in every bin according to the Bonferroni correction for multiple comparisons was calculated in the following manner: $p=\frac{0.01}{\mathrm{Nbins}}$. Assuming a normal distribution, we obtained the number of standard deviations (*Z*-value) required to attain the probability *p* and drew the lower and upper confidence levels at the ordinates corresponding to the mean ± *Z* standard deviations.

### Silence index

The increase in CV indicated that under anesthesia neuronal firing could not be modeled by a Poisson distribution. Therefore to enable comparison between neuronal relative quiescence periods while taking into account the different firing rates we created the Silence Index which normalized the relative quiescence time of a neuron with that expected from a Poisson neuron having the same firing rate. The proportion of time taken by the longest 5% of the ISIs relative to the whole recording time was calculated for the real neuron and its Poisson model neuron. Then the value of the real neuron was divided by that of its Poisson model. Values substantially higher than 1 indicate that the neuron tended to be more silent than a Poisson neuron with the same firing rate, suggesting that the real neuron compensated its prolonged silent periods by a higher firing rate during non-quiescent periods in order to obtain a similar firing rate.

## Results

**Figure 1:**
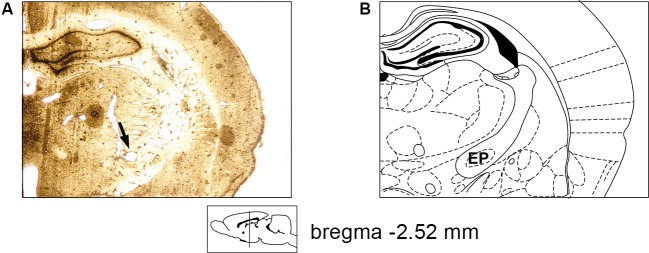
**Electrode placement verification. (A)** A 60 micron slice showing electrode positioning in the rat EP following electrolytic lesion (marked by an arrow). **(B)** Appropriate coronal section from the rat atlas (Bregma: −2.52 mm; Paxinos, 2007).

We recorded the activity of 38 single neurons in the EP of four rats and characterized their waveforms and firing parameters during different levels of isoflurane anesthesia and wakefulness. The electrode placement in the EP was verified histologically by making an electrolytic lesion via the electrodes that displayed neuronal activity during recording sessions (see Section Materials and Methods). An example of the placement of an implanted electrode in the EP is shown in Figure 1A. Its corresponding coronal slice taken from a rat atlas (Paxinos, 2007) is shown in Figure 1B. In each session neuronal activity was recorded during five states: four levels of isoflurane anesthesia (1%, 2%, 3% and 4%), and wakefulness, during which the animal could move freely in the recording chamber. Neuronal activity was recorded throughout each session with a 5 min intermission following each transition in anesthesia depth, thus allowing neuronal activity to physiologically adjust to the different levels (see Section Materials and Methods). Overall, in each state we analyzed neuronal activity for an average time of 511 ± 24.47 s.

**Figure 2:**
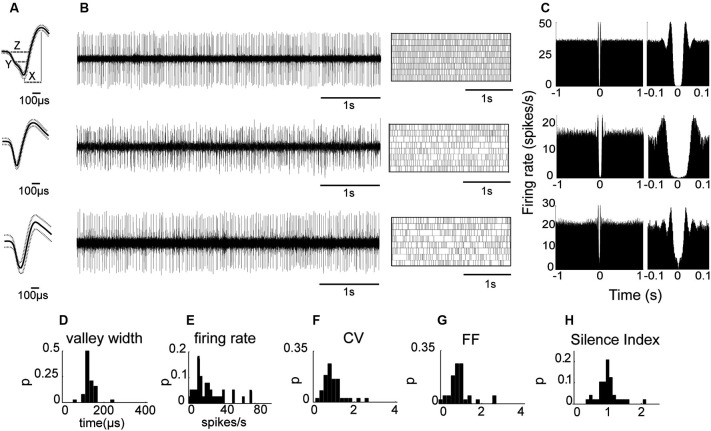
**EP neuronal firing characteristics in awake rats. (A)** Examples of the firing of three EP neurons. From left to right: Average waveform. Inset (top): *X* represents the valley to peak duration, *Y* the valley width and *Z* the zero-cross parameter. **(B)** Continuous raw signal during 5 s (left) and spike train during 20 s (right). **(C)** Autocorrelation using a time window of ±1 s (left) and autocorrelation with *x*-axis expanded to 0.1 s (right) of the same neuron. **(D–H)** Waveform and firing parameter histograms.

We first analyzed neuronal activity in the awake state to test whether as in primates the properties of the recorded population are uniformly distributed despite the possibility of including two subtypes as suggested by anatomical studies (Nakanishi et al., 1990; Parent et al., 2001). Initial observations indicated that all the recorded neurons displayed similar waveform shapes composed of a sharp narrow valley that was followed by a slightly longer duration peak. Waveform shapes of three EP neuronal exemplars are shown in Figure 2A. The waveforms were characterized by the following parameters described in the Materials and Methods section (see also inset in Figure 2A): the valley width at half amplitude (142 ± 4.43 μs), the waveform duration measured from the first valley to the following peak (258 ± 11 μs), the valley to peak amplitude ratio (0.67 ± 0.03), and the zero-crossing duration (275 ± 12 μs). Additionally, the neurons had an average refractory period of 6.97 ± 4.61 ms (mean ± std). Examination of the firing patterns displayed by EP neurons showed that these neurons fired tonically at a relatively high rate (26.0 ± 3.1 spikes/s; Figure 2B). Their firing was irregular as reflected in the relatively flat autocorrelograms over more than 1 s (see examples in Figure 2C). Of the 24 neuronal pairs recorded simultaneously during wakefulness, none showed a peak in the cross-correlogram suggesting that EP neurons did not interact with one another or that their interactions were negligible. Like their waveforms, EP neurons displayed homogenous firing patterns characterized by the following parameters: CV = 1.08 ± 0.08, FF = 1.02 ± 0.09, mode ISI = 19.2 ± 1.5 ms, and a Silence Index = 1.06 ± 0.05. The distributions of these parameters are shown in Figures 2D–H. Overall, the relatively narrow distributions with a single peak characteristic of these firing pattern statistics suggest that the recorded population was composed of a single cell type that fired in a Poissonian manner most of the time.

**Figure 3:**
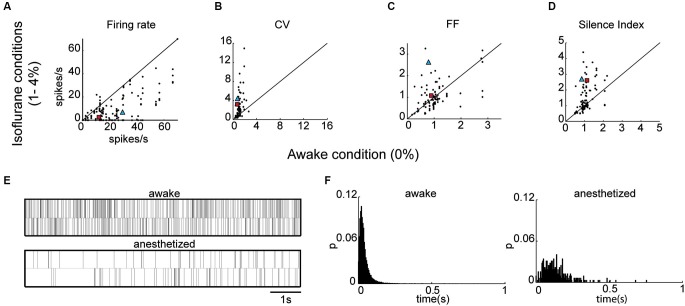
**EP neuronal firing characteristics in anesthetized rats. (A)** Firing rate under anesthesia vs. firing rate during the awake state. **(B–D)** Same as **A** for CV **(B)**, FF (**C**) and Silence Index **(D)**. **(E)** Representative spike train examples over 20 s of the corresponding neuron marked by a blue triangle in **A–D**. **(F)** ISI histograms of the corresponding neuron marked by a red rectangle in **A**–**D**.

We then examined the effect of isoflurane anesthesia on the properties of the EP neurons. The isoflurane did not influence the waveform shapes of the neurons, and all the measured parameters remained unchanged (valley width: *p* = 0.27, valley to peak duration: *p* = 0.94, zero-cross *p* = 0.62, valley to peak amplitude ratio: *p* = 0.97) indicating that the recordings were stable throughout the sessions. By contrast, firing patterns varied substantially with isoflurane administration (see Table 1). The firing rate decreased significantly during anesthesia (14.8 ± 1.5 spikes/s) relative to wakefulness (26.0 ± 3.1 spike/s; Kruskal Wallis; *p* = 0.00009) as shown in Figure 3A and in the example shown below of neuronal firing recorded during wakefulness and under anesthesia (Figure 3E). Additionally, the CV values were substantially higher during anesthesia (2.40 ± 0.28) relative to wakefulness (1.08 ± 0.08; *p* = 0.03; Figure 3B). The increase in the CV values combined with the decrease in FR suggested that EP neurons ceased firing for durations longer than expected from irregular Poisson firing. Indeed, the Silence Index measured during isoflurane administration (1.51 ± 0.09) was substantially higher than during wakefulness (1.06 ± 0.05; Figure 3D; *p* = 0.048). An example of the ISI histogram of one EP neuron is shown during anesthesia and wakefulness and illustrates the long tail of ISI observed under anesthesia (Figure 3F). The FF values within the population did not change with isoflurane administration (Figure 3C; *p* = 0.75), and the neuronal pairwise interactions (*n* = 24) continued to be void.

**Table 1:**
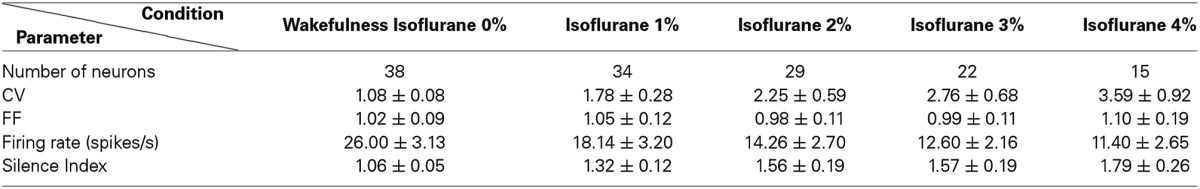
**Firing parameters across conditions (mean ± sem)**.

**Figure 4:**
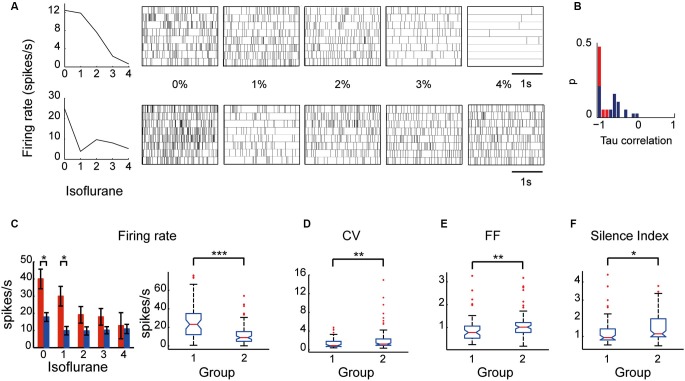
**Firing properties of EP neuronal subpopulations: Group I and Group II. (A)** An example of two neurons—one that decreased its firing rate correlatively with the increase in isoflurane level (top) and another that decreased its firing rate abruptly with isoflurane administration (bottom)—and their corresponding spike trains over 20 s. **(B)** Tau correlation histogram: red represents the neurons identified as displaying a correlated firing rate to isoflurane levels by the Kendall Tau correlation test. **(C)** (left) Average firing rate of the two groups (Group I is shown in red and Group II is shown in blue) during five states of anesthesia. (right) The boxplots depict the firing rate medians of the two groups under all conditions with the edges at the 25th and 75th percentiles. Vertical lines mark the 5th and the 95th percentiles. Red asterisks mark outliers. **(D–F)** Boxplots of CV **(D)**, FF **(E)**, and Silence Index **(F)** of the two groups under all conditions (* *p* < 0.05; ** *p* < 0.01; *** *p* < 0.001).

Examination of the relationship between the neuronal firing rate and the different levels of anesthesia revealed two types of EP neurons: one type (Group I) whose firing rate decreased as the anesthesia level increased, and a second type (Group II) whose firing rate was uncorrelated with anesthesia level. Neuronal spike trains representative of the two groups are shown in Figure 4A. The a-parametric Kendall Tau correlation test which identifies significant nonlinear correlations (*p* < 0.05) was used to determine which of the neurons decreased its firing rate with the increase in isoflurane level (see Section Materials and Methods). Observation of the Tau correlation factor showed that none of the neurons increased its firing rate with anesthesia (Tau values < 0); however, only those that substantially and monotonically decreased firing rate (values ~ −1) were identified as significant (red color; Figure 4B). Similar results were obtained using the Spearman’s *ρ* rank correlation test. Group I comprised 37% (14/38) of the EP neurons whereas Group II comprised the remaining 63% (24/38) of the recorded population. Statistical examination of the influence of isoflurane administration on the two groups revealed a main effect for both group type and anesthesia level (a two-way ANOVA with Tukey-Kramer correction for multiple comparisons; *p* < 0.0001). *Post-hoc* examination of the firing rate showed that during wakefulness Group I displayed a significantly higher firing rate than Group II. Moreover, the firing rate of Group I gradually decreased and reached significance at isoflurane level of 2% relative to wakefulness whereas the firing rate of Group II decreased abruptly with anesthesia administration and remained constant during all isoflurane levels (Figure 4C).

Taking into consideration that neurons with similar firing rates can display different firing patterns and *vice versa* neurons with different firing rates can display similar firing patterns we tested whether the two groups display similar firing pattern characteristics. Specifically, we statistically compared the values of the firing pattern parameters CV, FF and silence index between the two groups which seemed non-separable in the awake state. Although both groups significantly decreased their firing rate in response to anesthesia they were characterized by different firing pattern parameters (Figures 4D–F). Group I displayed lower values of CV (1.70 ± 0.34), Silence Index (1.26 ± 0.11), and FF (0.91 ± 0.08) compared to Group II (CV: 2.25 ± 0.27; *p* = 0.004; Silence Index: 1.47 ± 0.08, *p* = 0.014; and FF: 1.09 ± 0.06, *p* = 0.008). These data suggest that the two groups may reflect the existence of two subpopulations of neurons in the EP that could only be distinguished by their characteristic electrophysiological properties during different levels of anesthesia.

## Discussion

In this study we characterized neuronal activity in the EP of anesthetized and freely moving rats. Our results show that in freely moving animals, EP neurons displayed a high firing rate of 26 ± 3.1 spikes/s (range: 2.23–76.13 spikes/s, median: 20.30 spikes/s) whereas administration of isoflurane anesthesia induced a significant decrease in the firing rate to an average of 14.8 ± 1.5 spikes/s (range: 0.2–74.4 spikes/s, median: 9.27 spikes/s). This decrease in firing rate occurred either gradually as more anesthesia was delivered, or abruptly with the transition between wakefulness and the first level of anesthesia and then remained relatively stable regardless of the anesthesia level. Based on this distinction in the neurons’ responses to anesthesia, we classified the EP neurons into two groups: Group I and Group II. Although examination of the firing properties of EP neurons during wakefulness indicated a homogenous population characterized by near Poissonian tonic firing and a lack of interactions, further examination suggested otherwise; the newly defined groups had significantly different firing pattern properties. Our results provide evidence for the potential existence of two distinct subpopulations within the rat EP that could be distinguished by their electrophysiological characteristics during isoflurane anesthesia.

Analysis of EP neurons recorded during wakefulness without a-priori classifying them into two distinct groups based on their anesthesia response curves revealed a seemingly homogeneous population. Similarly, extracellular recordings in the primate GPi revealed one homogenous neuronal population (DeLong, 1971). The firing pattern characteristics of GPi neurons excluding firing rate described in later studies by DeLong (1971) and others (Joshua et al., 2009; Sheth et al., 2011) are similar in nature to those observed in this study. Additionally, EP neurons were uncorrelated and apparently did not interact with one another as was previously observed in primates (Bar-Gad et al., 2003a), strengthening the view that the BG act as a decorrelating and dimensionality reduction system (Bar-Gad et al., 2003b). Under isoflurane, EP neurons increased their CV and Silence Index and decreased FR indicating that in that condition these neurons displayed a higher firing variability and pauses longer than expected from a Poissonian firing. A similar result was obtained in humans under Propofol anesthesia which inhibited GPi neurons, and in addition to inducing long pauses, increased their burst index (Hutchison et al., 2003).

The main difference between the neuronal activity of EP neurons and that of the GPi neurons is their average firing rate, which was 26.0 ± 3.1 spikes/s in EP and 63 spikes/s in GPi (DeLong, 1971). In a previous study we characterized neuronal activity of GP neurons in freely moving rats and compared the results to those obtained in primate GPe neurons using the same methodology (Benhamou et al., 2012). The outcome of that study showed that similarly to primates, rat GP neurons could be divided into two distinct neuronal populations based on their characteristic firing patterns. Two measures diverged from primates: the first was the average firing rate that appeared to be much lower in rats (20.07 spikes/s) than in primates (55 spikes/s), and the second was that the two subpopulations represented different proportions (rats: 73.5 vs. 26.5%; primates: 85 vs. 15%, for high frequency pausers and low frequency bursters, respectively (DeLong, 1971; Bugaysen et al., 2010; Benhamou et al., 2012). It should be noted that the proportions of these subpopulations resembled the *in vitro* proportions reported in rodents (Kita and Kitai, 1991; Cooper and Stanford, 2000). The current results further support the view that firing patterns rather than firing rates and subpopulation proportions are important to the preservation of BG information processing and especially the GP and EP, and are therefore conserved across species.

Isoflurane administration substantially altered the firing patterns displayed by EP neurons. Isoflurane is minimally metabolized in the body and has a low blood-gas partition coefficient (1.4) so that it has a very low solubility in blood and changes in concentrations are rapid. In the present experiment, we estimated a recovery time of 5 min based on our observations of the time it took animals to walk normally in the recording chamber following the removal of 1% isoflurane anesthesia. Recently, a median recovery time of 330 s has been reported when animals were placed under 1.5% isoflurane for 40 min (Taylor et al., 2013). Therefore, we cannot rule out that some of the anesthesia evoked effects may have had a longer lasting influence on the electrophysiological characteristics of EP neurons. Such a case however would have likely minimized rather than enhanced or caused the observed differences between the two groups because of residual effect averaging.

*In vitro* studies in rodents, cats and primates have reported the presence of two neuronal populations based either on their biophysical properties (Nakanishi et al., 1990) or on their projections’ target sites (Nauta, 1974; Filion and Harnois, 1978; van der Kooy and Carter, 1981; Tokuno et al., 1988; Parent et al., 2001). On one hand, a proportion of 2/3 to 1/3 was found in rats based on the EP projections to the LHb and to the thalamus, respectively (van der Kooy and Carter, 1981). On the other hand, when the identification of two types of neurons was based on their membrane electrical properties a different proportion of division into types was reported; namely, 79.6% of type I neurons and 16.6% of type II neurons (Nakanishi et al., 1990). By taking into account that the intracellular recording method may create a sampling bias and consequently skew the data toward more accessible neurons, it is plausible that despite the difference in proportions the two studies dealt with the same neuronal types (i.e., that EP neurons projecting to different targets may be biophysically distinct). Isoflurane operates by altering neuronal membrane excitability (Franks and Lieb, 1994; Arhem et al., 2003) suggesting that neurons with different membrane properties may display different response curves under anesthesia. Therefore, based on the sensitivity of the membrane excitability to isoflurane and the similarity between the reported proportions of the two identified neuronal populations and the proportions observed in this study (63% in Group II and 37% in Group I) it is possible that the neurons comprising Group I are those projecting to the thalamus and those comprising Group II project to the LHb.

A complementary factor which may accentuate the differences between the firing properties of EP neurons is the different origin of their inputs; pallidohabenular neurons receive afferents from striatofugal neurons in the patch compartment whereas pallidothalamic neurons receive afferents from the matrix compartment (Rajakumar et al., 1993). It remains unknown however whether medium spiny neurons in the patch compartment respond to anesthesia differently than medium spiny neurons in the matrix. Another possible partition of EP neurons into subpopulations is based on their neurotransmitters; pallidothalamic neurons seem to comprise mainly GABAergic projections whereas the majority of the pallidohabenular projections in rats are non-GABAergic (Araki et al., 1984), and are possibly cholinergic (Moriizumi and Hattori, 1992). However, the release of different neurotransmitters is less likely to account for the observed differences in the groups’ response curves and would yield a different group ratio (~1:1; GABAergic vs. non-GABAergic neurons) than the observed ratio (2:1; pallidohabenular vs. pallidothalamic). Therefore, a classification of Group I and Group II as GABAergic and possibly cholinergic neurons is less probable.

Primate and cat pallidohabenular neurons, which are far less abundant (10 and 16% of the neurons, respectively) than rat pallidohabenular neurons (2/3 of the neurons), have lower baseline firing rates (33 spikes/s) than presumed motor GPi neurons (77 spikes/s) (Larsen and Mcbride, 1979; Hong and Hikosaka, 2008). In the current study we found that 63% of the neurons (Group II neurons) had significantly lower firing rates than Group I neurons in awake rats (Group II: 17.8 ± 2.5 spikes/s; and Group I: 40.0 ± 5.7 spikes/s). The similarity in the observed firing rate imbalance between the two identified groups in rats and between the habenular projecting GPi neurons and motor neurons in primates lends additional support to the hypothesis that the Group II neurons reported in this study are habenular projecting EP neurons whereas the Group I neurons are thalamus projecting EP neurons. By taking into account the small size of rat EP in the rostro-caudal axis (600–800 μm) it seems that confirmation of this hypothesis could be obtained by optogenetic activation of identified habenular projecting EP neurons or by their antidromic activation via stimulation of the LHb. If confirmed, the simple procedure of isoflurane administration or possibly other kinds of anesthesia would permit selective access to limbic vs. motor information processing in the rat EP and primate GPi.

## Conclusion

Our results suggest that: (1) firing patterns are preserved in the rat EP compared to primate GPi whereas firing rate and subpopulation ratio are not; and (2) the use of isoflurane anesthesia may provide an easy method to accentuate electrophysiological differences in the EP neurons and consequently enable their classification into two distinct neuronal populations. In so doing our results potentially reconcile *in vitro* studies performed in cat and rodent EP, and primate GPi where two subpopulations of neurons have been identified, and electrophysiological studies in behaving primates where only one homogeneous neuronal population has been reported. Further experiments are required to determine whether these two populations of neurons correspond to pallidohabenular and pallidothalamic neurons, and investigate whether their information processing capacity and contribution to behavior are similar or different in nature.

## Conflict of interest statement

The authors declare that the research was conducted in the absence of any commercial or financial relationships that could be construed as a potential conflict of interest. The Associate Editor, Dr. Alon Korngreen declares that, despite being affiliated to the same institution as the authors, the review process was handled objectively and no conflict of interest exists.
